# Tumor-induced remote ECM network orientation steers angiogenesis

**DOI:** 10.1038/srep22580

**Published:** 2016-03-02

**Authors:** Hayri E. Balcioglu, Bob van de Water, Erik H. J. Danen

**Affiliations:** 1 Division of Toxicology, Leiden Academic Center for Drug Research, Leiden University, Leiden, the Netherlands

## Abstract

Tumor angiogenesis promotes tumor growth and metastasis. Here, we use automated sequential microprinting of tumor and endothelial cells in extracellular matrix (ECM) scaffolds to study its mechanical aspects. Quantitative reflection microscopy shows that tumor spheroids induce radial orientation of the surrounding collagen fiber network up to a distance of five times their radius. Across a panel of ~20 different human tumor cell lines, remote collagen orientation is correlated with local tumor cell migration behavior. Tumor induced collagen orientation requires contractility but is remarkably resistant to depletion of collagen-binding integrins. Microvascular endothelial cells undergo directional migration towards tumor spheroids once they are within the tumor-oriented collagen fiber network. Laser ablation experiments indicate that an intact physical connection of the oriented network with the tumor spheroid is required for mechanical sensing by the endothelial cells. Together our findings indicate that, in conjunction with described activities of soluble angiogenic factors, remote physical manipulation of the ECM network by the tumor can help to steer angiogenesis.

Tumor-associated angiogenesis is one of the hallmarks of cancer^1^,^2^. The chemotactic aspect of this pathology has been well studied. Oncogenic signaling pathways and hypoxia occurring in tumors activate the release of growth factors, such as vascular endothelial growth factor (VEGF), which triggers the formation of new microvascular sprouts from pre-existing vessels. Inhibitors against this paracrine interaction, targeting mainly the VEGF receptor (VEGFR) on endothelial cells, have entered the clinic^3^,^4^.

Angiogenesis involves proliferation and migration of endothelial cells^5^. During angiogenesis, endothelial cells migrate through a 3D extracellular matrix (ECM) network that is rich in collagen. Migration efficiency and the mode of migration (e.g. the extent of integrin-dependency and the requirement of matrix metalloproteases) are determined by ECM properties including ligand density, stiffness, fiber crosslinking, and pore size^6^,^7^,^8^. These ECM properties are typically altered in tumor areas; e.g. ECM stiffening has been observed in tumor tissue^9^,^10^.

The ECM network may control angiogenesis in several ways. First, it acts as an organizing platform for growth factor distribution, activation, and presentation^11^. *In vitro* assays have shown that tissue deformation can regulate angiogenesis through spatial organization of activity of the VEGF pathway^12^. Additionally, cells receive mechanical cues from the ECM through integrin-based cell-matrix adhesions^13^,^14^,^15^. *In vivo* studies have demonstrated that angiogenesis is an integral response to chemical and mechanical cues^16^.

Here we use sequential microprinting of tumor and microvascular endothelial cells to investigate their mechanical interaction through ECM scaffolds. We use quantitative reflection microscopy analysis to study tumor-induced collagen orientation. We show that tumor spheroids can orient a fibrillar collagen network to a distance of up to 5 times the tumor radius – far beyond the area of tumor expansion and cell migration. Furthermore, we demonstrate that microvascular endothelial cells sense and respond to such orientation provided that the oriented ECM is physically connected to the tumor spheroid. Together, our data indicates that ECM network reorientation acts as a remote mechanical cue to steer angiogenesis.

## Results

### Tumor spheroids in 3D collagen induce the orientation of surrounding collagen

**Figure 1:**
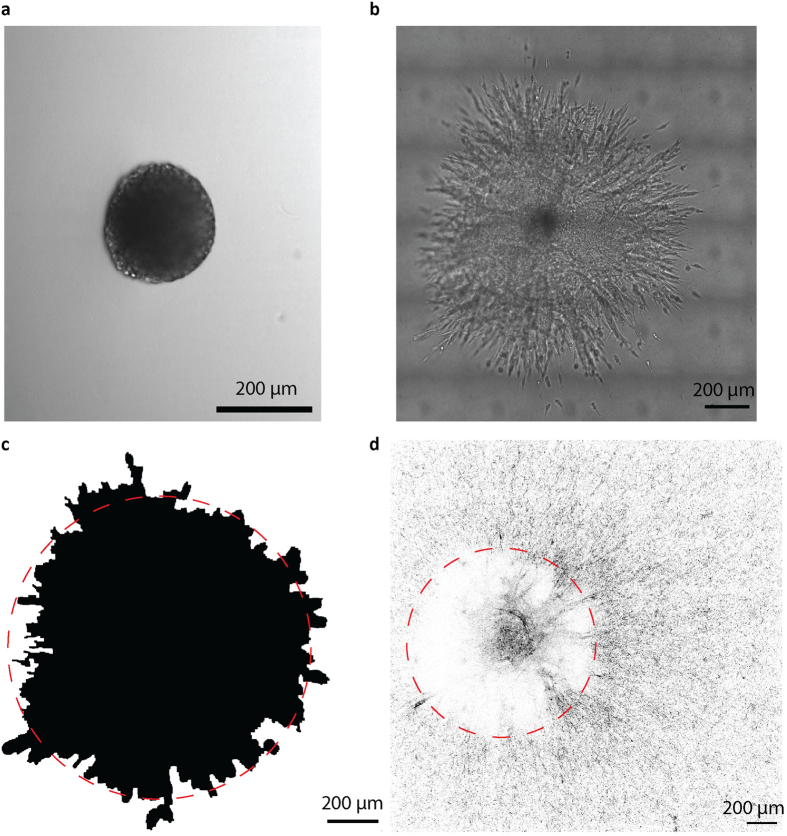
4T1 breast cancer spheroid expansion and collagen network organization. (**a,b**) 4T1 tumor spheroid at the day of injection (**a**) and 48 hours after injection (**b**). (**c**) Spheroid mask covering core spheroid and migrating cells at 48 hours, which was used as boundary for collagen organization calculations (red dashed circle). (**d**) Inverted reflection microscopy image with tumor border (red dashed circle) showing radial orientation of surrounding collagen.

**Figure 2:**
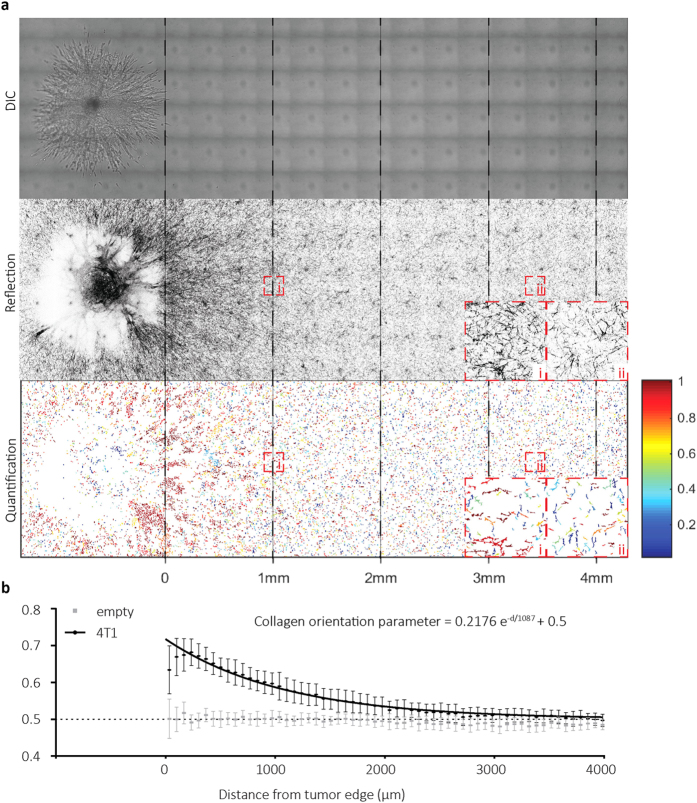
4T1 breast cancer spheroids cause long distance radial orientation of surrounding collagen. (**a**) DIC (top) and reflection microscopy (middle) images of collagen-embedded spheroid 48 hours post injection and corresponding collagen orientation detection (bottom; red, radially oriented collagen fibers; blue, tangential collagen fibers). Dashed lines note the indicated distances from tumor spheroid border. Note the dense red at distance 0–1 mm with gradually increasing randomness of colors at increasing distances. In i and ii zoom-ins of the indicated areas are shown. (**b**) Collagen orientation parameter calculated 48 hours after injection at the indicated distances from individual tumor spheroid borders for 29 4T1 injections (black circles) and 22 wells lacking tumor spheroids (gray squares) from 5 independent experimental replicas with standard deviations. The fit equation (black line) is shown.

4T1 breast cancer spheroids were generated by cell microprinting in collagen gels (Fig. 1a) and their outgrowth and migration was monitored after 48 hours (Fig. 1b). A spheroid mask was generated to define the final spheroid area including spheroid core and migrated cells (Fig. 1c). Reflection microscopy was performed to analyze the collagen network surrounding this final spheroid area (Fig. 1d). In 2 days the tumor spheroid radius (including core and migrated cells) increased ~4-fold (Fig. 1a,b). Concomitantly, reflection microscopy showed a denser fibrillar collagen network surrounding the tumor expansion region (Figs 1d and 2a). Quantitative image analysis showed an increase in collagen fibers with an orientation parameter ~1 (dark red; denoting collagen directed radially towards the tumor spheroid center) close to the spheroid boundary (Fig. 2a). By contrast, in areas distant from the spheroid, the number of fibers with an orientation parameter ~1 equaled the number of fibers with an orientation parameter ~0 (dark blue; denoting collagen oriented tangential to the tumor spheroid radius) (Fig. 2a). Quantification of collagen fiber orientation throughout the gel relative to the distance from the final tumor spheroid edge (dashed red circle in Fig. 1c,d) indicated that tumor spheroids that expanded from an average radius of 116 ± 21 μm to 527 ± 54 μm had caused radial orientation of collagen fibers up to 2.65 mm from the spheroid edge (i.e. 95% confidence interval >0.5 indicating orientation was significantly different from random) (Fig. 2b). Thus, tumor spheroids induced remote orientation of collagen fibers up to distances of 5 times the spheroid radius.

### Remote tumor-induced collagen network reorientation correlates with local cell migration capacity and requires Rho kinase-myosin activity

**Figure 3:**
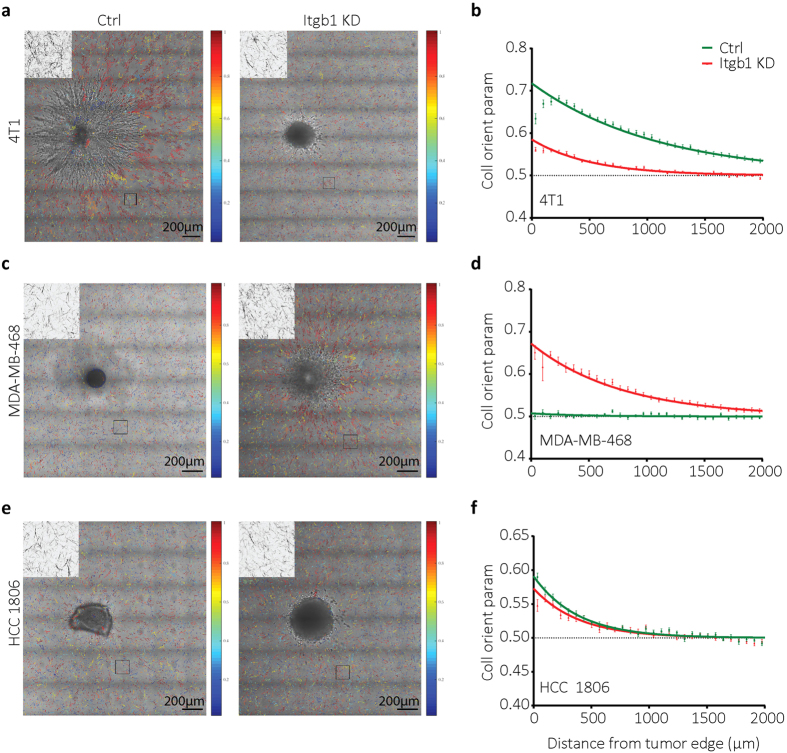
Distinct effects of β1 integrin downregulation on tumor spheroid cell migration and collagen orientation. (**a,c,e**) Collagen orientation images merged with DIC images taken 48 hours after injecting the indicated cell lines with or without shRNA targeting *ITGB1*. Collagen image zoom-ins of the indicated black boxes 500 μm from the tumor spheroid edge are shown at left top of each image. (**b,d,f**) Collagen orientation measured at a range of distances from tumor border for 4T1 shctrl (b, green; n = 29), 4T1 shITGB1 (b, red; n = 29), MDA-MB-468 WT (d, green; n = 16), MDA-MB-468 shITGB1 (d, red; n = 21), HCC1806 shctrl (f, green; n = 20), and HCC1806 shITGB1 (f, red; n = 21) tumor spheroids 48 hours after injection. Mean ± standard deviation with exponential fits (solid lines) from at least four independent experimental replicas is shown.

To address the role of collagen-binding integrins (mainly α1β1, α2β1) in tumor-induced collagen orientation we made use of cells stably expressing shRNAs targeting *ITGB1*, causing strongly reduced (~90%) levels of β1 integrins (Supplementary Fig. S1)^17^. For 4T1 cells, depletion of β1 integrins reduced spheroid expansion through collective migration and caused a switch to individual cell migration as described before^17^ (Fig. 3a). This was accompanied by reduced collagen orientation (measured beyond the area of cell migration) (Fig. 3a). Similar results were obtained for HCC70-derived tumor spheroids (Supplementary Fig. S1). By contrast, control MDA-MB-468 and BT20 tumor spheroids showed little migration whereas depletion of β1 integrins in these cells enhanced spheroid expansion through a mix of collective and single cell migration (Fig. 3c and Supplementary Fig. S1). In these cases, depletion of β1 integrins led to an increased remote collagen orientation (Fig. 3d and Supplementary Fig. S1). Lastly, in HCC1806 cells β1 integrin depletion caused a shift from relatively ineffective collective migration to similarly weak single cell migration and this did not affect the capacity of the tumor spheroids to cause collagen orientation (Fig. 3e,f). Together, these results indicated that the capacity of tumor cells to orient the collagen network was not affected by a reduction in collagen-binding integrins per se. Rather, changes in integrin expression caused decreased or increased tumor cell migration at the spheroid edge, which correlated with decreased or increased remote collagen orientation capacity, respectively.

**Figure 4:**
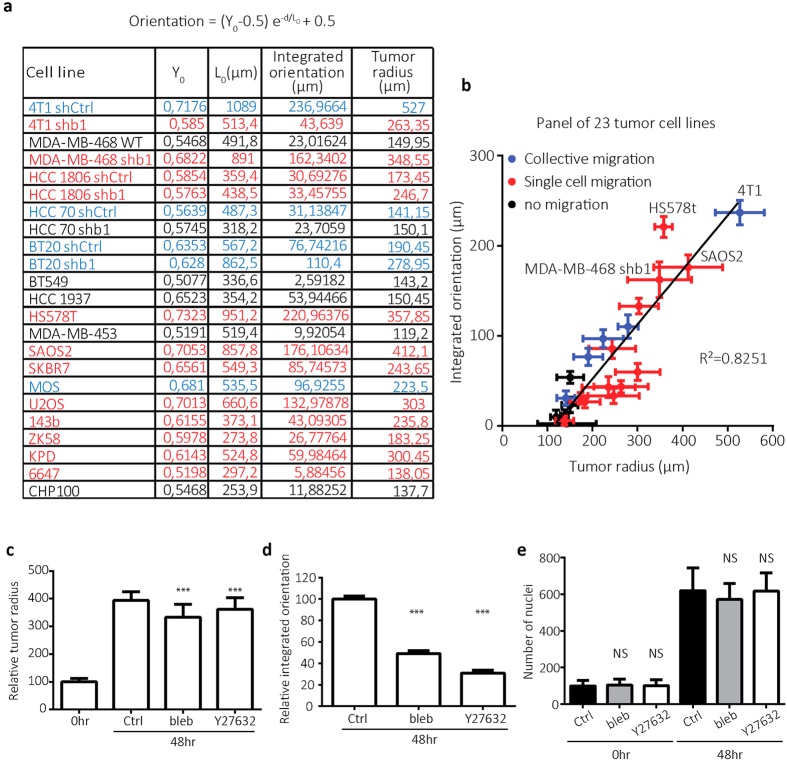
Tumor induced collagen orientation requires ROCK-myosin II-induced contractility. (**a**) Table showing the fit parameters Y_0_ and L_0_, the area under the fitted curve (integrated orientation) and the tumor radius 48 hours after injection for indicated cell lines. (**b**) Graph showing relation between tumor radius at 48 hours post injection and integrated collagen orientation parameter for cell lines depicted in table a with distinct modes of migration as indicated based on DIC images. Plot shows mean, standard deviation and linear fit (Y = 0.605( ± 0.06)X-68.65( ± 15.6); R^2^ = 0.8251). (**c,-e**) Bar graphs showing mean and standard deviation of 4T1 tumor radius 48 h post injection normalized to spheroid size directly after injection (0 h) (**c**), integrated collagen orientation at 48 hours normalized to control (**d**), and number of nuclei at 0 and 48 h after injection (**e**) for no treatment (Ctrl), 10 μM blebbistatin (bleb) and 10 μM Y27632 (Y27632). NS; p > 0.05 and ***p < 0.0005 according to Mann-Whitney test (**c,e**) or unpaired t-test (**d**) compared to control.

We next tested a larger panel of carcinoma and sarcoma cell lines for their capacity to orient surrounding collagen (Supplementary Fig. S2). In line with the results obtained with 4T1 cells, irrespective of the origin of the cell line, β1 integrin expression level, or migration strategy; there was a strong correlation between remote collagen orientation capacity and spheroid expansion (average initial spheroid radius for all cell lines was 113 ± 29 μm, x-intercept of linear fit was 113 ± 26 μm) (Fig. 4a,b). Measured spheroid expansion included spheroid growth and migration. Tumor cell types showing the largest spheroid expansions typically displayed strong migration activity. To investigate the role of cytoskeletal contractility, pharmacological inhibition of myosin II or Rho kinase that acts upstream of myosin II activity was used for the duration of the experiment. Treatment of 4T1 spheroids with a myosin II inhibitor caused a ~15% decrease in final spheroid radius and reduced collective migration activity that was accompanied by a 50% reduction in remote collagen orientation (Fig. 4c,d and Supplementary Fig. S3). Inhibition of Rho kinase led to a ~8% decrease in final spheroid radius and caused a switch from collective migration to individual cell migration that was accompanied by a 70% reduction in remote collagen orientation (Fig. 4c,d and Supplementary Fig. S3). Neither Blebbistatin nor Y-27632 at the concentrations used affected cell proliferation (Fig. 4e and Supplementary Fig. S3). These results showed that inhibition of Rho kinase-myosin II-mediated contractility has moderate effects on spheroid expansion but strongly attenuates remote collagen orientation.

### Tumor-oriented collagen network directs endothelial cell migration

**Figure 5:**
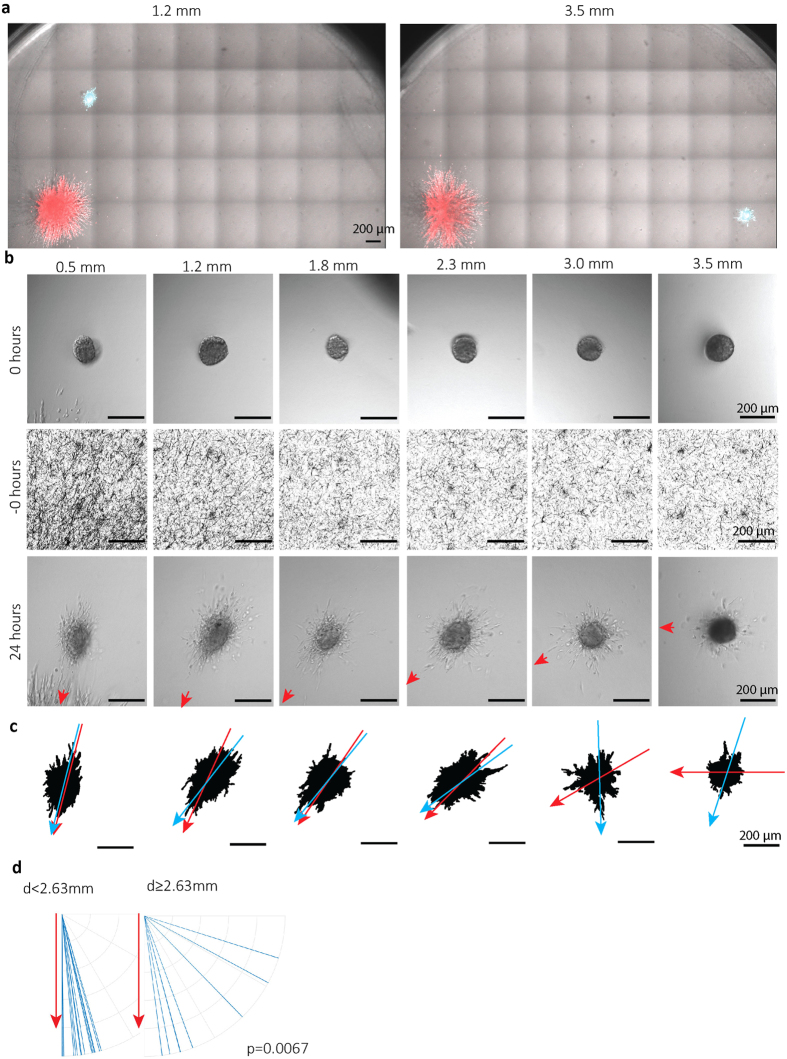
HMEC-1 microvascular endothelial cells injected within 4T1 remote area of oriented collagen show directional migration towards tumor spheroid. (**a**) Merged DIC/fluorescence images taken at t = 72 hours showing CellTracker green CMFDA-labeled HMEC-1 cells injected at t = 48 hours at the indicated distances from the spheroid border of CellTracker Orange CMRA-labeled 4T1 cells injected at t = 0. (**b**) Representative DIC images of HMEC-1 spheroids at the day of injection (top) or 24 hours after injection (bottom) at indicated distances from 4T1 tumor spheroids, and reflection microscopy images showing collagen network at the same location prior to the injection (middle). The red arrows point towards the center of 4T1 tumor spheroid. (**c**) HMEC-1 spheroid masks generated for images shown in b (bottom). Blue arrow indicates major axis of the mask; red arrow points to 4T1 tumor spheroid center. (**d**) Major axis orientation of HMEC-1 spheroids (blue lines) injected at distances from 4T1 spheroid edge less (n = 15; left) or more than 2.63 mm (n = 8; right) plotted against the direction of the 4T1 tumor spheroid (set vertically for each experiment; red arrow). Data obtained from four independent experiments; P value calculated using Mann-Whitney test.

HMEC-1 human microvascular endothelial cells were injected at various defined x-y distances from 4T1 spheroids at 48 hours post 4T1 injection (Fig. 5a and Supplementary Fig. S4). The resulting endothelial spheroids were monitored by DIC, 24 hours later and the direction of the tumor spheroid was marked (red arrow head) (Fig. 5b). Endothelial spheroid masks were generated to study the orientation of their long axis (Fig. 5c; blue arrows) and relate this to the relative position of the tumor spheroid (Fig. 5c; red arrows). Alignment of the endothelial spheroid in the direction of the tumor spheroid was observed for injections within the 2.65 mm collagen orientation area whereas this was lost for endothelial cells injected beyond this zone (Fig. 5d).

**Figure 6:**
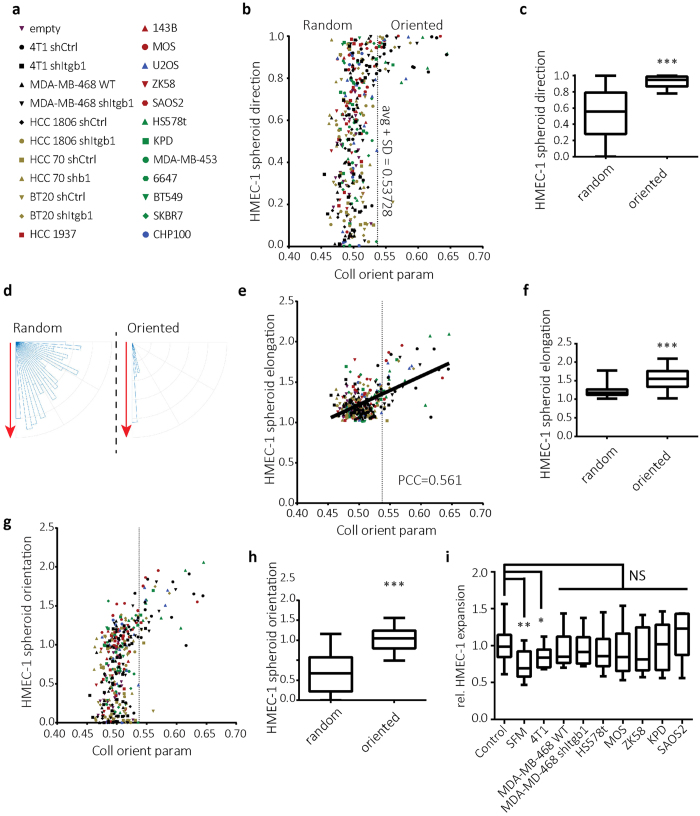
Directional HMEC-1 migration towards tumor spheroid when injected within area of tumor-oriented collagen for panel of tumor cell lines. (**a**) Panel of cell lines used with corresponding symbols. (**b,e,g**) Direction (**b**) elongation (**e**) and orientation (**g**) of HMEC-1 spheroids measured 24 hours after HMEC-1 injection at varying distances from panel of tumor spheroids (**a**) plotted against collagen orientation parameter at the corresponding distances (obtained in each case from reflection microscopy 48 hours after tumor cell injection just prior to HMEC-1 injection). Dashed line is drawn at one standard deviation above average collagen orientation parameter and indicates the threshold for “oriented collagen”. (**c,f,h**) Box whisker graphs showing the minimum to maximum of direction (**c**) elongation (**f**) and orientation (**h**) of HMEC-1 spheroids injected in regions of oriented collagen vs regions of random collagen. (**d**) Major axis direction of HMEC-1 injections in oriented collagen vs random collagen plotted against the direction of the tumor spheroids (set vertically for each experiment; red arrow). (**i**) Box whisker graph showing HMEC-1 spheroid expansion following 24 hours incubation with conditioned medium from the indicated tumor cell lines normalized to complete HMEC-1 culture medium (Control); SFM, serum free MCDB131 medium. NS, p > 0.05, *p < 0.05, **p < 0.005, ***p < 0.0005 according to Mann-Whitney test. PCC: calculated Pearson product-moment correlation coefficient.

Next, HMEC-1 cells were injected at varying distances from tumor spheroids derived from the panel of cell lines described above (Fig. 6a). The distance to which HMEC-1 cells could sense and respond to these spheroids differed in accordance with the large variation in collagen orientation distance among these lines (Fig. 4). Across the panel of cell lines, HMEC-1 spheroids present within a zone of strong tumor-oriented collagen were directed towards the tumor spheroid whereas direction of HMEC-1 spheroids was random if they were outside of this zone (Fig. 6b–d). HMEC-1 directionality was induced in response to collagen orientation significantly above average as measured for all tested HMEC-1 injection coordinates (Fig. 6b). This level of orientation was reached by 4T1, HCC70, Hs578t, SAOS2, U20S, MOS and KPD but not BT20 or MDA-MB-468 cells. In accordance with the data shown above (Fig. 3; Supplemental Fig. S1) β1 integrin-depletion reduced above-threshold collagen orientation measurements for 4T1 and HCC70 cells whereas this was induced for BT20 and MDA-MB-468 cells in response to *ITGB1* silencing. Likewise, HMEC-1 spheroid elongation correlated with tumor-induced collagen orientation (Fig. 6e,f) and combining HMEC-1 spheroid direction and elongation parameters showed a strong and significant HMEC orientation response to tumor-oriented collagen (Fig. 6g,h). To rule out differences in the production of soluble angiogenic factors as the underlying cause for different efficiencies in triggering HMEC-1 orientation, we tested the effects of conditioned media derived from the tumor cell lines used. None of the conditioned media, whether obtained from highly effective (e.g. 4T1, SAOS2) or ineffective tumor cell lines (e.g. KPD, MDA-MB-468) induced HMEC-1 migration above the level observed with standard complete HMEC-1 culture medium (Fig. 6i; Supplementary Fig. S4).

### Endothelial response to oriented collagen network requires physical coupling with tumor

**Figure 7:**
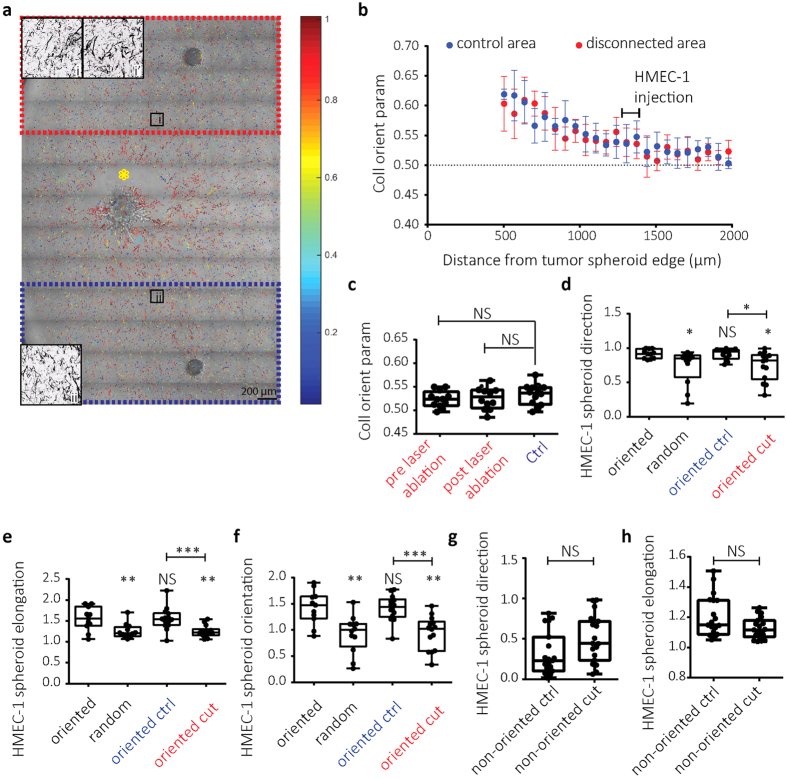
Endothelial response to tumor-oriented collagen network depends on physical connection of oriented collagen network with the tumor. (**a**) Collagen orientation image superimposed on DIC image showing 4T1 spheroid (center) and two HMEC-1 spheroids at the top and bottom of the image at ~1.4 mm from the 4T1 injection. Yellow asterisk indicates area of laser severing 48 hours post 4T1 injection just after HMEC-1 injection; red box, area disconnected from the tumor; blue box, control area. In i and ii collagen images for zoom-ins of the indicated areas are shown. i’ is the image obtained from the same location as i prior to laser severing. (**b**) Collagen orientation parameter (mean ± standard deviation) for control (blue) and disconnected (red) areas. The region where HMEC-1 cells were injected is indicated. (**c**) Box whisker graphs showing the minimum to maximum of collagen orientation parameter at the HMEC-1 injection sites just prior to HMEC-1 injection for blue box in Fig. a (Ctrl) and red box in Fig. a before and after laser ablation. (**d–h**) Box whisker graphs showing the minimum to maximum of direction (**d,g**), elongation (**e,h**) and orientation (**f**) of HMEC-1 spheroids injected in oriented or random collagen regions 48 hours after 4T1 injections (black legends; data for 4T1 cells from Fig. 5) or injected in the blue box (oriented ctrl) or the disconnected red box (oriented cut) as indicated in a (**d–f**) or in absence of tumor cells (**g,h**) 24 hours after HMEC-1 injection and subsequent laser ablation. NS, p > 0.05; *p < 0.05; **p < 0.01; ***p < 0.001 relative to oriented unless otherwise indicated; Mann-Whitney test.

To address if physical connections between the tumor spheroid and the oriented collagen network remained important for guidance of endothelial cells, the spheroid was physically disconnected after the collagen network had been oriented. For this purpose, two HMEC-1 spheroids were injected at the same distance from the tumor spheroid at opposite sides and laser severing of collagen fibers was applied close to the tumor edge, between the tumor and one of the HMEC-1 spheroids (Fig. 7a). Orientation of the collagen network was maintained in areas disconnected from the tumor spheroid through laser severing (Fig. 7b,c). However, HMEC-1 cells injected in such areas no longer responded to collagen orientation: HMEC-1 spheroid direction, elongation, and the combined orientation parameter were decreased; resembling HMEC-1 behavior in non-oriented collagen areas (Fig. 7d–f). Control HMEC-1 spheroids in the same well that were still connected to the tumor normally responded to oriented collagen (Fig. 7d–f). As a control, laser ablation in non-oriented collagen did not affect the random HMEC-1 migration (Fig. 7g,h and Supplementary Fig. S5). These findings demonstrate that an intact physical connection of the oriented ECM network with the tumor spheroid is required for orientation sensing by the endothelial cells.

## Discussion

The tumor stroma plays an important role in initiation and progression of cancer^18^. Mechanical properties of the ECM can influence tumor cell behavior and have been linked to prognosis. ECM stiffness^19^,^20^,^21^, pore size^22^,^23^,^24^, crosslinking^25^, fiber alignment^26^,^27^, as well as the presence of stromal contractile cells^28^ have all been shown to influence aspects of cancer progression, including tumor growth and invasion. Vice-versa, tumor cells actively modify these ECM properties thereby promoting tumor growth, invasion, and metastasis potential^29^,^30^,^31^,^32^.

Here, we use quantitative reflection microscopy analysis to study remote tumor-mediated collagen network orientation. We show that tumor spheroids reorient a surrounding collagen-based ECM network up to five times their radius. In a panel of cell lines the distance of collagen orientation correlates with spheroid expansion, which is driven by local invasion/migration. Such long range collagen reorganization has also been observed for mouse fibroblast explants^33^. Local ECM reorganization in areas containg tumor cells is driven by Rho kinase-Myosin II-mediated contractility^19^,^34^,^35^. Our findings indicate that traction forces applied by the tumor cells on the local collagen network drives ECM reorientation also in distant areas where tumor cells are absent. The consequences of contractility inhibition for local tumor cell migration are limited. This may be explained by tumor cell plasticity: tumor cells can switch to modes of migration that are less dependent on contractility^6^,^7^,^8^. By contrast, the capacity of a tumor to cause remote ECM reorientation is strongly attenuated under conditions of reduced contractility.

Antibody blocking experiments have shown that collagen-binding integrins mediate i) local tumor-induced collagen network reorganization^36^, and ii) tumor cell-responses to mechanical ECM properties^25^. Gene silencing as used in our study may be less efficient than antibody blocking. Nevertheless, we observe highly distinct effects of β1 integrin silencing on collagen network reorientation. We and others have previously shown that depletion or blockade of β1 integrins can either inhibit migration or cause a switch from collective to single cell migration, e.g. through effects on TGF-β signaling^17^,^37^,^38^. Our current study shows that in tumors where collective migration is attenuated or switched to less abundant individual cell migration in response to β1 integrin silencing, collagen network reorientation is lost (e.g. 4T1); whereas in tumors where cell motility is normally very poor and β1 integrin silencing triggers more abundant (individual) cell migration, a concomitant increase in collagen network reorientation is observed (e.g. MDA-MB-468). The fact that β1 integrin silencing does not directly attenuate collagen organization may point to roles for other collagen-binding receptors. On stromal fibroblasts, syndecan-1 participates in ECM network alignment^39^. Likewise, syndecans or discoidin domain collagen receptors on tumor cells may be candidates for force-induced collagen reorganization in the context of strongly reduced integrin levels. Moreover, αvβ3 that is present on MDA-MB-468 and induced on 4T1 cells in response to β1 integrin silencing, may be involved through binding cryptic RGD sites in areas where collagen is processesed by tumor cells^17^,^40^

Our findings indicate that tumor spheroids can reorient collagen networks at relatively long distances, way beyond the area of tumor expansion and migration. Endothelial cells can sense such long-range orientation and respond by moving towards the tumor. It is known that mechanical ECM properties, such as density and stiffness regulate angiogenesis^41^,^42^,^43^,^44^. This may be explained by changes in the distribution of soluble factors or enhanced activity of the receptors for these factors^12^,^45^,^46^. Alternatively, physical aspects of the network may instruct endothelial cell behavior. Indeed, we show that tumor-mediated remote radial organization of collagen directs human microvascular endothelial cells. The correlation between levels of remote collagen organization and induction of endothelial cell directionality holds through for a panel of ~20 different human cancer cell lines. Laser severing of collagen fibers close to the tumor does not affect the architecture of the remote collagen network but leads to complete loss of endothelial cell responsiveness to such oriented ECM regions. This indicates that contact guidance, i.e. a preference for aligned collagen fibers, is insufficient. Rather, once the collagen network is organized, distant forces applied to the network by the tumor are critical for sensing and/or responding of endothelial cells.

We show that conditioned media from the tumor cell lines used do not enhance migration capacity of the endothelial cells beyond the level of migration observed in the presence of complete medium, as used in our experimental system. Together with the laser severing experiment, this argues against a mechanism involving different levels of secretion of soluble angiogenic factors as the underlying cause for the different efficiencies with which the tumor cell lines control endothelial cell migration. Importantly, our study does not oppose the established role for soluble factors in tumor angiogenesis. In our experiments, soluble factors potentially modulating HMEC-1 migration are present in the HMEC-1 medium in a spatially random manner. Under those conditions, collagen network orientation by the tumor spheroid directs endothelial cell migration. *In vivo*, distant ECM reorganization may act in synergy with paracrine signals produced by the tumor to direct microvascular sprouts towards the tumor.

Taken together, our study shows for the first time that a radial collagen network organization generated by the tumor relatively far beyond the area of tumor expansion and migration, not only forms migratory highways for tumor invasion but can also guide angiogenesis in a manner dependent on tumor generated traction forces. In coordination with gradients of soluble factors and matrix remodeling by cells in the tumor microenvironment such as cancer-associated fibroblasts, this mechanical interaction might further direct microvascular sprouts towards the tumor. Hence, targeting tumor induced ECM remodeling may prevent both tumor invasion and angiogenesis.

## Materials and Methods

### Cell culture

4T1 mouse breast cancer cells and BT20, BT549, HCC1806, HCC1937, HCC70, HS578t, MDA-MB-453, MDA-MB-468, and SKBR7 human breast cancer cells purchased from the American Type Culture Collection or provided by Dr. J Foekens, Erasmus Medical Center, Rotterdam NL^47^ were grown in RPMI1640 medium supplemented with 10% fetal bovine serum (GIBCO, USA), 25 U/ml penicillin and 25 μg/ml streptomycin (Invitrogen). Human osteosarcoma cell lines MOS, U2OS, 143B, ZK58, SAOS2, and KPD were described previously^48^ and grown in the same medium. Human Ewing sarcoma cell lines 6647 and CHP100 were provided by Dr. P. Hogendoorn, Leiden University Medical Center, Leiden NL, and maintained in IMDM cell culture medium (GIBCO) supplemented with 10% fetal bovine serum, 25 U/ml penicillin and 25 μg/ml streptomycin. Stable bulk-sorted *ITGB1*-silenced tumor cell lines were described previously^17^. HMEC-1 human microvascular endothelial cells^49^ were cultured in MCDB131 medium (GIBCO) supplemented with 15% fetal bovine serum, 200 mM L-Glutamine, 10 μg/mL epidermal growth factor, 100 μg/mL hydrocortisone, 25 U/ml penicillin and 25 μg/ml streptomycin. All cells were cultured in a humidified incubator at 37 °C with 5% CO_2_.

### Automated sequential microprinting of tumor- and endothelial cells in ECM scaffolds

Collagen type I solution was isolated from rat-tail collagen by acid extraction as described previously^50^. Collagen was diluted to 1 mg/mL in the culture medium containing 0.1 M Hepes (BioSolve) and fixed to pH 7.5 by addition of NaHCO_3_ (stock 440 mM, Merck). 60 μL of this solution was then pipetted into a glass-bottom 96 well plate (Greiner) and incubated for 1 hour at 37 °C to polymerize.

Automated injection of cell suspensions into the resulting collagen gels to generate arrays of cell spheroids with defined x-y-z position was performed as described, using injection robotics from Life Science Methods, Leiden NL (http://www.lifesciencemethods.com)^17^,^51^. Tumor spheroids of 113 ± 29 μm initial radius were generated at 200 μm above the glass surface (average collagen gel height ~1.5 mm) and incubated 48 hours with appropriate culture media for each cell line. Subsequently, medium was removed, HMEC-1 cells were injected at the same z-position at various defined x-y distances from the tumor spheroid, and wells were further incubated with HMEC-1 culture media for 24 hours (Supplementary Fig. S4).

For experiments where tumor spheroids were treated with Myosin II or Rho kinase inhibitors, media was supplemented with blebbistatin (Calbiochem cat. number 203389, Merck KGaA, Darmstadt, Germany) or Y27632 (Tocris cat. number 1254, Bristol, UK), respectively reaching 10 μM final concentration (medium + gel). For fluorescent imaging of 4T1 and HMEC-1, cells were incubated at 37 °C with 1 μM CellTracker Orange CMRA or CellTracker Green CMFDA Dye, respectively for 15 minutes prior to injection; or with 1 μg/mL Hoechst 33342.

For experiments where HMEC-1 spheroids were incubated with conditioned media from tumor cell lines, 100 μL of supernatant from 48 hours of cell culture of indicated cell lines where cells were seeded at ~30% confluency and reached ~95% confluency at the day of extraction, was added on top of the collagen gel following the injection of HMEC-1 cells. For controls 100 μL complete HMEC-1 culture medium as used throughout all other experiments, or MCDB131 medium supplemented with 25 U/ml penicillin and 25 μg/ml streptomycin was used. Images were acquired and expansion was calculated after 24 hours.

### Collagen gel imaging

Spheroids were imaged using a Nikon TE2000 confocal microscope equipped with a Prior stage controlled by NIS Element Software and with a temperature and CO_2_-controlled incubator. Frame stitching was used when necessary. Differential interference contrast (DIC) images were captured using a charged coupled device (CCD) camera with NIS software and 10× dry objective. Reflection microscopy of the entire well was performed by 5.4 mm × 5.4 mm stitching of images obtained using a 40× long distance water immersion objective by illuminating with a 561 laser coupled with a 561 blocking dichroic mirror for the detection.

### Laser severing assay

After injecting HMEC-1 cells on both sides of the tumor spheroid at a distance of 1650 μm from the tumor spheroid center, laser severing was performed on one side of the tumor spheroid. 16 lines/second stimulation was applied just outside of the tumor spheroid (~1 mm from the HMEC-1 injection site), with infrared laser (Coherent Chameleon Discovery) at 790 nm wavelength at full power (~3000 mW), using the 40× long distance water immersion lens, while manually scanning through the z plane until all fibers were severed. For control experiments addressing the effect of laser severing in random collagen networks, the same procedure was used at ~500 μm from the HMEC-1 injection site in absence of tumor spheroids.

### Image analysis

All image analysis was performed using in house written Matlab scripts (Mathworks, Natick, MA, USA). DIC images were first put through a median filter to create a background illumination signal to which the original images were normalized. Normalized images were blurred and a mask for core detection was generated by thresholding for signal lower than two standard deviations below the mean and taking only the central binary image. Subsequently, a canny edge detection method was applied to the normalized image to mask the outer rim of the spheroid. This mask was dilated to include the area of cell migration and combined with the core mask to capture the entire final spheroid.

For nuclei counting, the Hoechst signal obtained over multiple z-stacks spanning the full thickness of the injection was summed, passed through a Gaussian low pass filter, and signal that was two standard deviations larger than the average of the signal was identified as foreground. Individual nuclei were then identified following watershed algorithm.

For reflection image analysis, first a background image was calculated by applying a circular averaging filter of 10 pixel radius to the original image. This image was then subtracted from the original image and a customized rollingball filter was applied to extract fibrillar structures. The filter multiplied the signal with itself and used a local thresholding algorithm assigning pixels with squared intensities >0.5 standard deviations above mean squared intensity within 5 px distance, to a collagen fiber. From this binary image, isolated pixels were removed, a binary closure was performed, and structures of >20 pixels and eccentricity ≥0.9 were assigned as fibers.

The directionality of a fiber was quantified by first manually determining the center of the tumor spheroid per image, and subsequently calculating for each fiber; the cosine square of the angle between the vector pointing from the tumor spheroid to the center of the collagen fiber and the orientation of the collagen fiber. The distance of a fiber to the tumor edge was calculated by subtracting the previously determined tumor spheroid radius (obtained from the DIC image analysis) from the distance of the fiber center to the tumor spheroid center. Fiber orientations were analyzed depending on their distance, in bins of 100 px (67 μm). To this data a two-parameter single exponential plateauing at 0.5 was fitted with the equation 
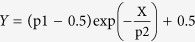
 for × (distance) larger than 100 μm using GraphPad Prism 6 program (GraphPad Software, La Jolla, CA). Integrated orientation was calculated from the fit by taking the integral 
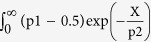
 d× which yielded the result (p1-0.5)*p2.

The collagen organization at the locations of HMEC-1 spheroids that were injected at designated distances from tumor center, was determined by quantifying the collagen organization at that distance from the tumor spheroid before the HMEC-1 injections were performed. When quantifying collagen orientation for the laser severing experiment the collagen organization was quantified both before HMEC-1 injection and after HMEC-1 injection/laser severing was performed. The HMEC-1 direction was determined by calculating the angle between the vector pointing from the HMEC-1 center to the tumor spheroid center and HMEC-1 long axis obtained from the injection mask, subtracting this angle from 90 degrees and dividing by 90 degrees so that HMEC-1 directed towards the tumor had a direction of 1 and HMEC-1 directed perpendicularly had a direction of 0. The elongation was calculated by dividing the long axis by the short axis length for the HMEC-1 spheroid mask. Pearson product-moment correlation coefficient and linear fit were obtained using GraphPad Prism 6 software. The spheroid orientation was calculated by multiplying the direction with the elongation parameter.

To calculate significance between two conditions, the Mann–Whitney U test was used when comparing distribution data, and unpaired t-test was used when comparing integrated collagen orientation.

## Additional Information

**How to cite this article**: Balcioglu, H. E. *et al.* Tumor-induced remote ECM network orientation steers angiogenesis. *Sci. Rep.* **6**, 22580; doi: 10.1038/srep22580 (2016).

## Supplementary Material

Supplementary Information
