# Supplementary material for: Tumor-induced remote ECM network orientation steers angiogenesis
Source: Sci Rep. 2016 Mar 2;6:22580. doi: 10.1038/srep22580 (PMC4773852; doi:10.1038/srep22580)
Supplement: Supplementary Information [file srep22580-s1.pdf]

Tumor-induced remote ECM network orientation steers angiogenesis

SUPPLEMENTARY INFORMATION

Hayri E Balcioglu[1], Bob van de Water[1] & Erik HJ Danen[1]

[1]Division of Toxicology, Leiden Academic Center for Drug Research, Leiden University, Leiden, the Netherlands

Corresponding authors: Hayri E Balcioglu, h.e.balcioglu@lacdr.leidenuniv.nl; Erik HJ Danen, e.danen@lacdr.leidenuniv.nl

Supplementary Figure S1. Effects of β1 integrin downregulation on tumor spheroid cell migration and collagen orientation. **(a,c,e)** Collagen orientation images merged with DIC images taken 48 hours after injecting the indicated cell lines with or without shRNA targeting *ITGB1* (a,c) or without injection (e). Collagen image zoom-ins of the indicated black boxes 500 µm from the tumor border are shown at left top of each image. **(b,d,f)** Collagen orientation measured at a range of distances from tumor border for HCC 70 shctrl (b, green; n=16), HCC 70 shITGB1 (b, red; n=15), BT20 shctrl (d, green; n=17), BT20 shITGB1 (d, red; n=17) tumor spheroids 48 hours after injection, and from the average injection location for empty well (f, black; n=22) at the same time point, mean ± standard deviation with exponential fits (solid lines) from at least three independent experimental replicas is shown. **(g)** Western blots showing integrin β1 and tubulin expression levels in indicated cell lines with or without shITGb1.

Supplementary Figure S2. Collagen organization and tumor expansion for a panel of cell lines. Collagen orientation images merged with DIC images taken 48 hours after injecting the indicated human breast cancer and sarcoma cells. Collagen image zoom-ins of the indicated black boxes 500 µm from the tumor border are shown at left top of each image. Graphs show corresponding collagen orientation measured at a range of distances from tumor border; mean ± standard deviation with exponential fits (solid lines) is shown.

Supplementary Figure S3. Effect of Y27632 and blebbistatin treatment on collagen organization. **(a-c)** Collagen orientation images merged with DIC images of 4T1 spheroids grown 48 hours in absence (a) or presence of 10µM Y27632 (b) or 10µM blebbistatin (c). Collagen image zoom-ins of the indicated black boxes 500 µm from the tumor border are shown at left top of each image. **(d)** Collagen orientation measured at a range of distances

from spheroid border for 4T1 injections after 48 hours without treatment (black, n=55), with 10µM Y27632 (green, n=46) or 10µM blebbistatin treatment (red, n=53), or measured from the average injection location in uninjected collagen gels (blue, n=23); mean ± standard deviation with exponential fits (solid lines) from three independent experimental replicas is shown. **(e)** sum of intensities z-projection images showing live Hoechst staining of 4T1 spheroids grown 48 hours in absence (left) or presence of 10µM Y27632 (middle) or 10µM blebbistatin (right).

Supplementary Figure S4. Automated sequential cell microprining layout for tumor spheroid-HMEC-1 interaction and effect of tumor cell secreted soluble factors on HMEC-1 migration. **(a)** Low magnification image of multiwell plate showing 4T1 cells (red arrow heads) injected at identical x-y-z position in each well followed by HMEC-1 cells (blue arrow heads) injected at varying distances 48 hours later and incubated for an additional 24 hours. **(b)** DIC image of HMEC-1 spheroids after 24 hours incubation with normal HMEC-1 cell culture media, serum free media or media extracted from indicated cell cultures.

Supplementary Figure S5. Laser severing does not affect endothelial cell migration towards it. Collagen orientation images (top) and corresponding DIC images (bottom) at the same location showing two HMEC-1 spheroids 48 hours after injection. Yellow asterisk indicates area of laser ablation performed just after HMEC-1 injection (t=0 h) (right). No laser ablation was performed for the HMEC-1 spheroid on the left.

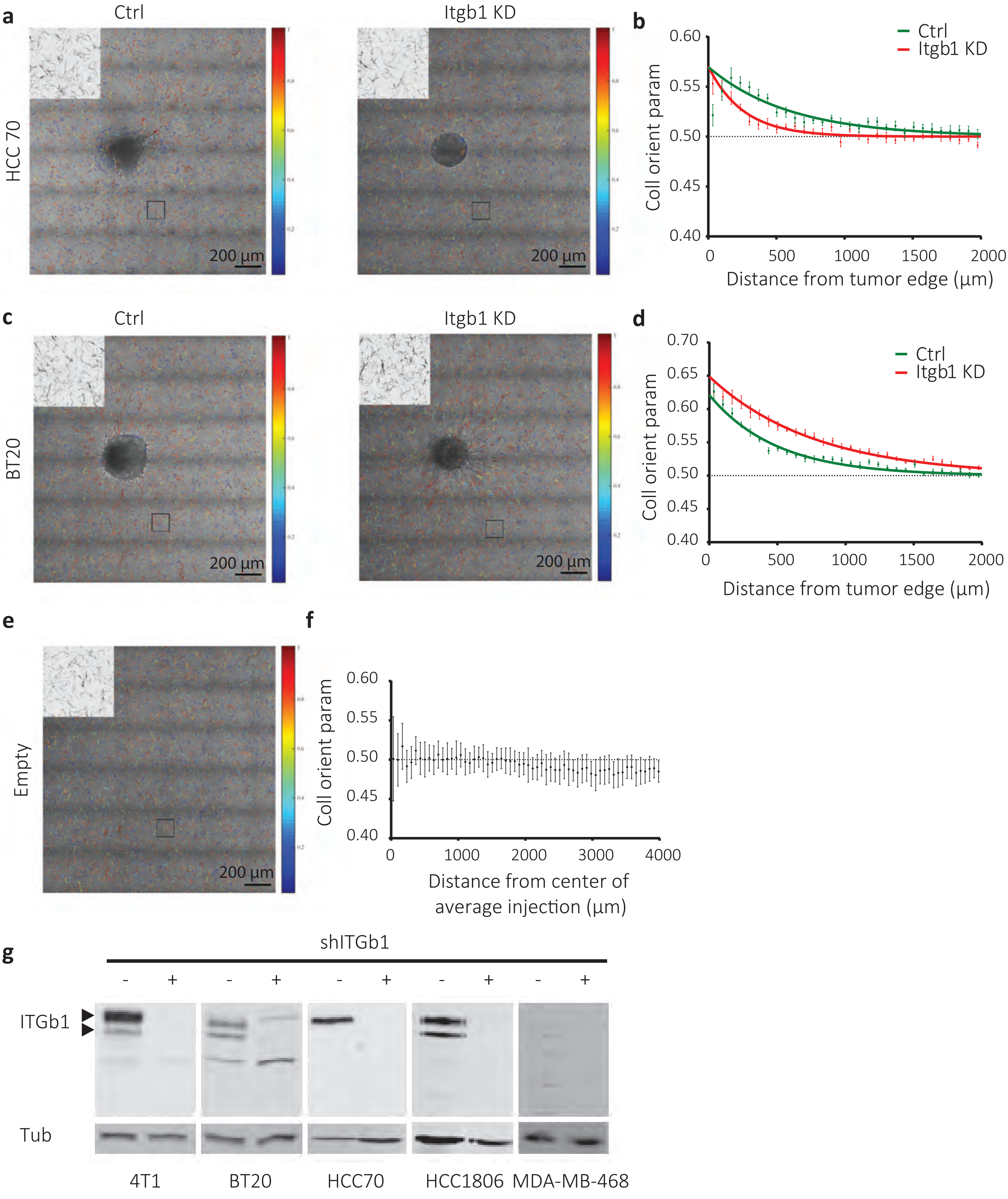

balcioglu supplementary figure 1

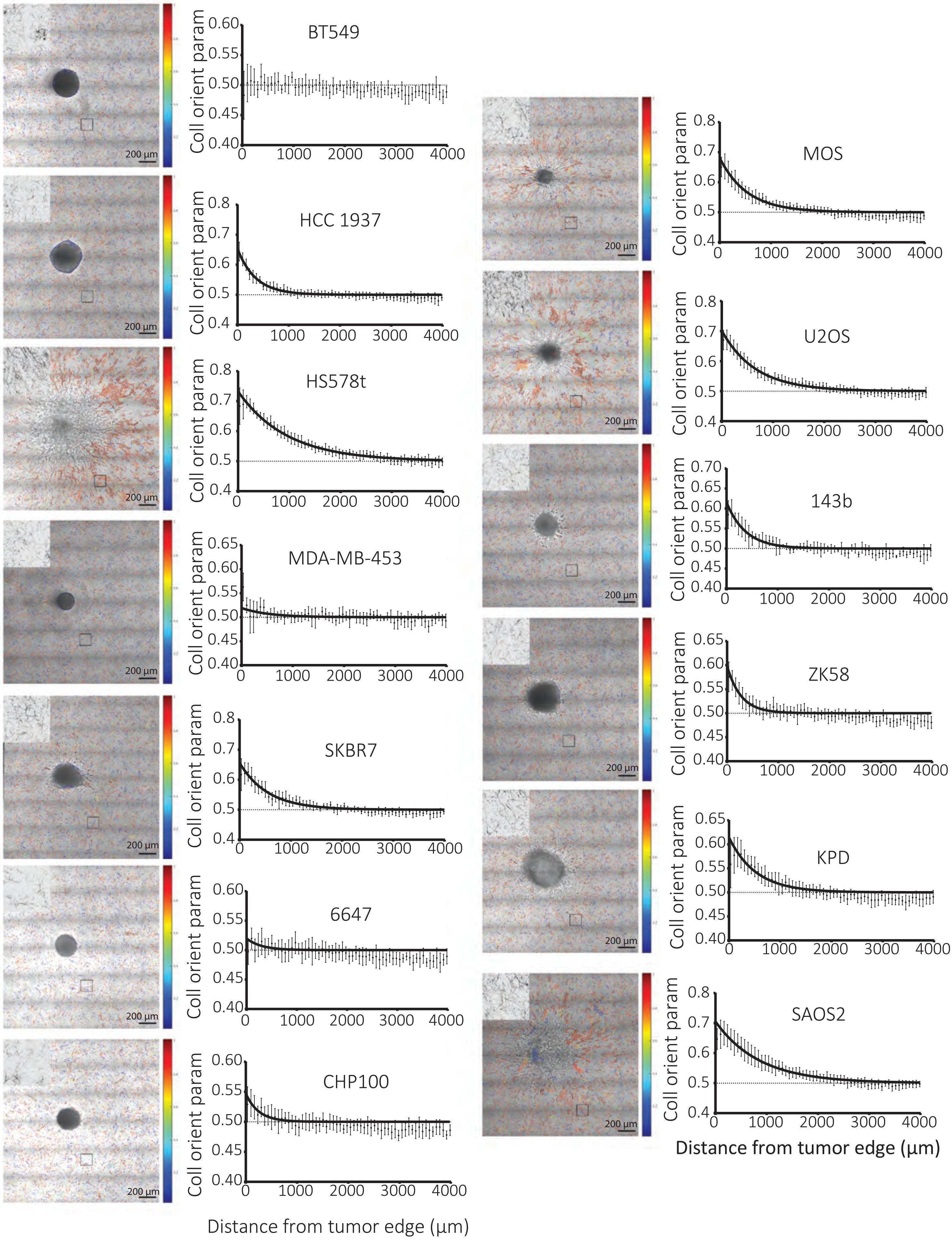

balcioglu supplementary figure S2

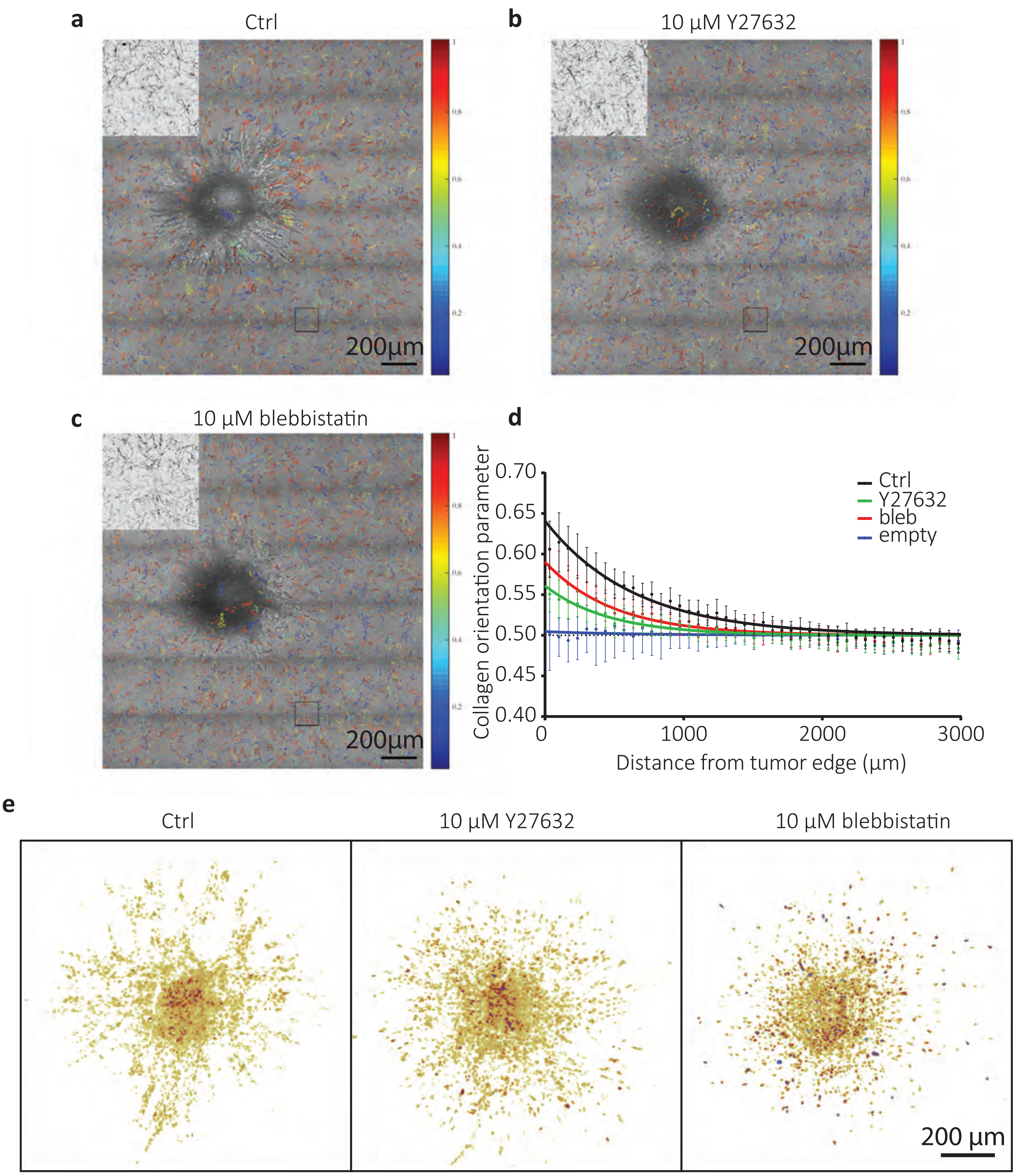

balcioglu supplementary figure S3

a

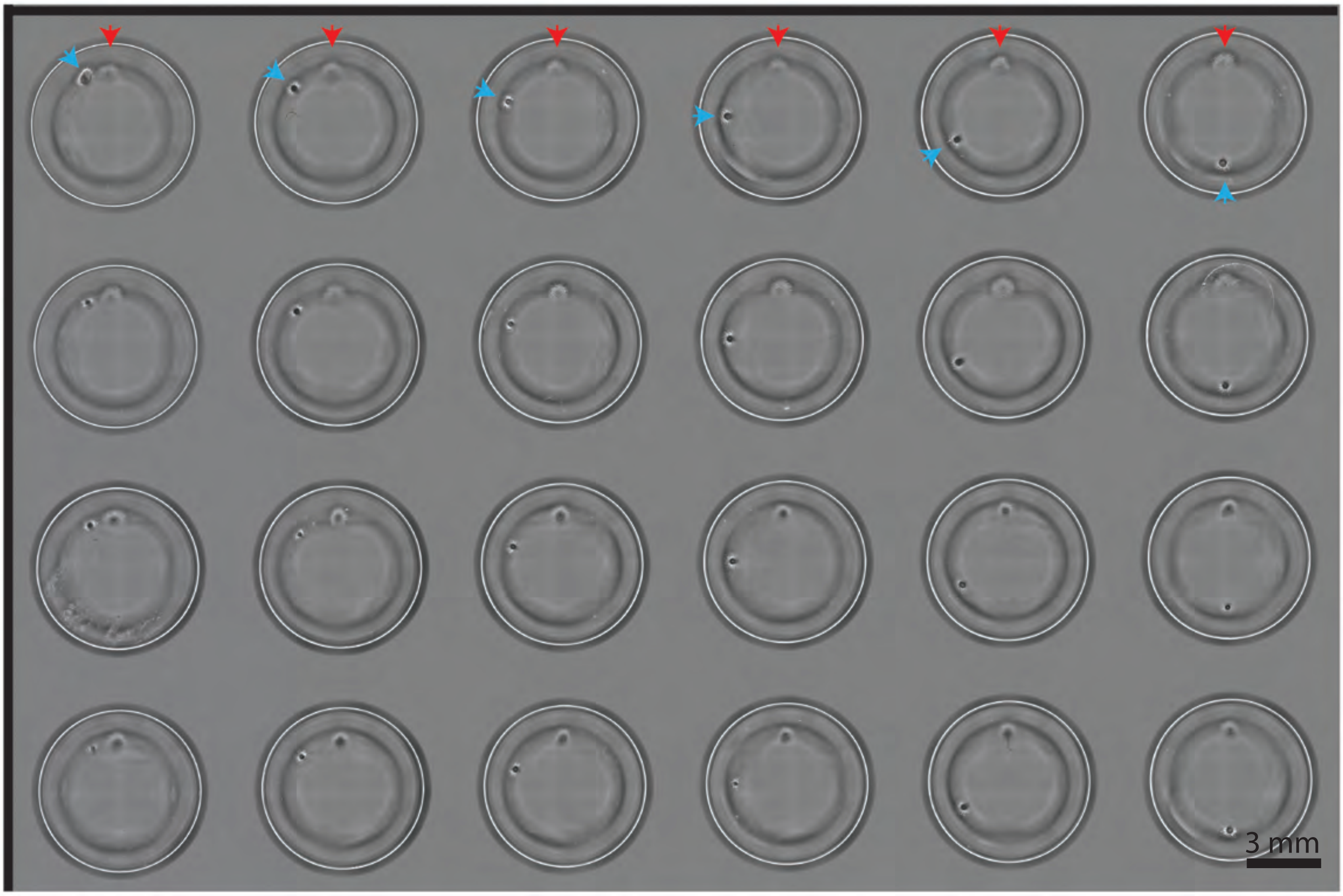

b

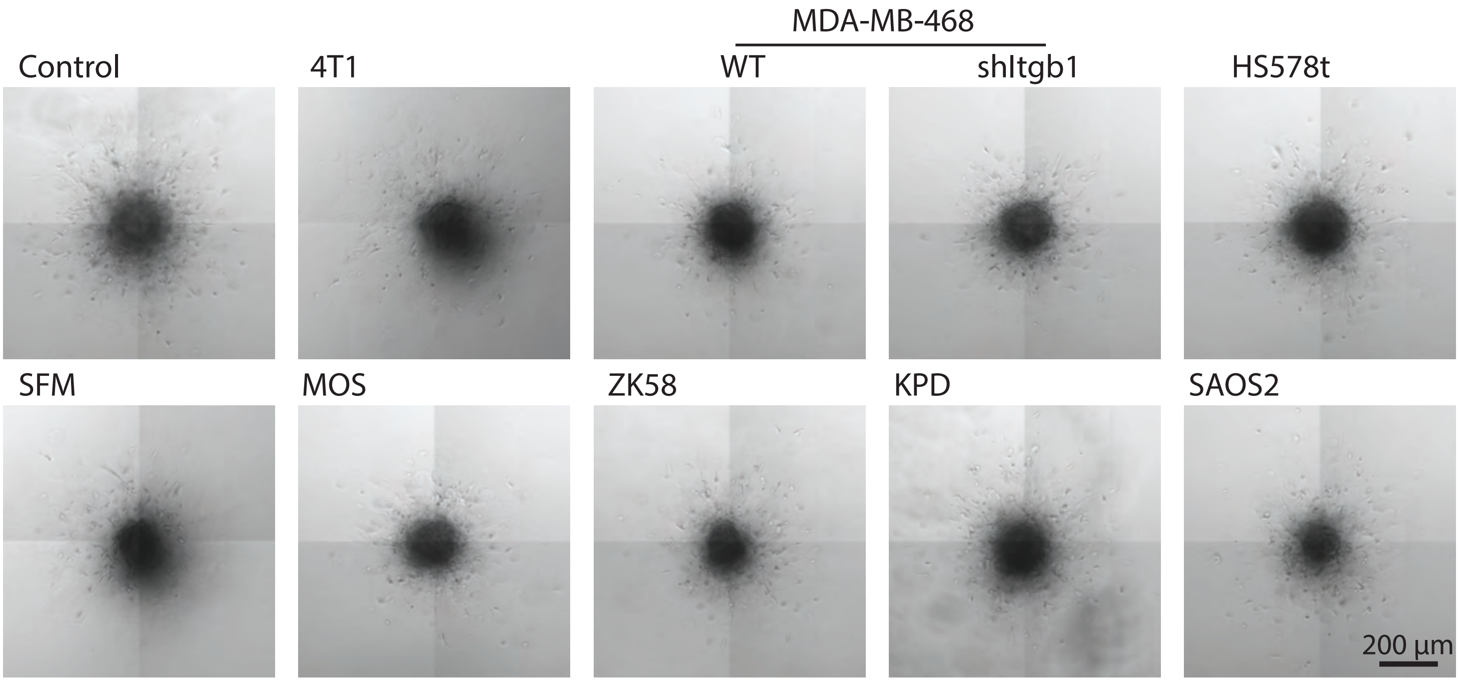

balcioglu supplementary figure S4

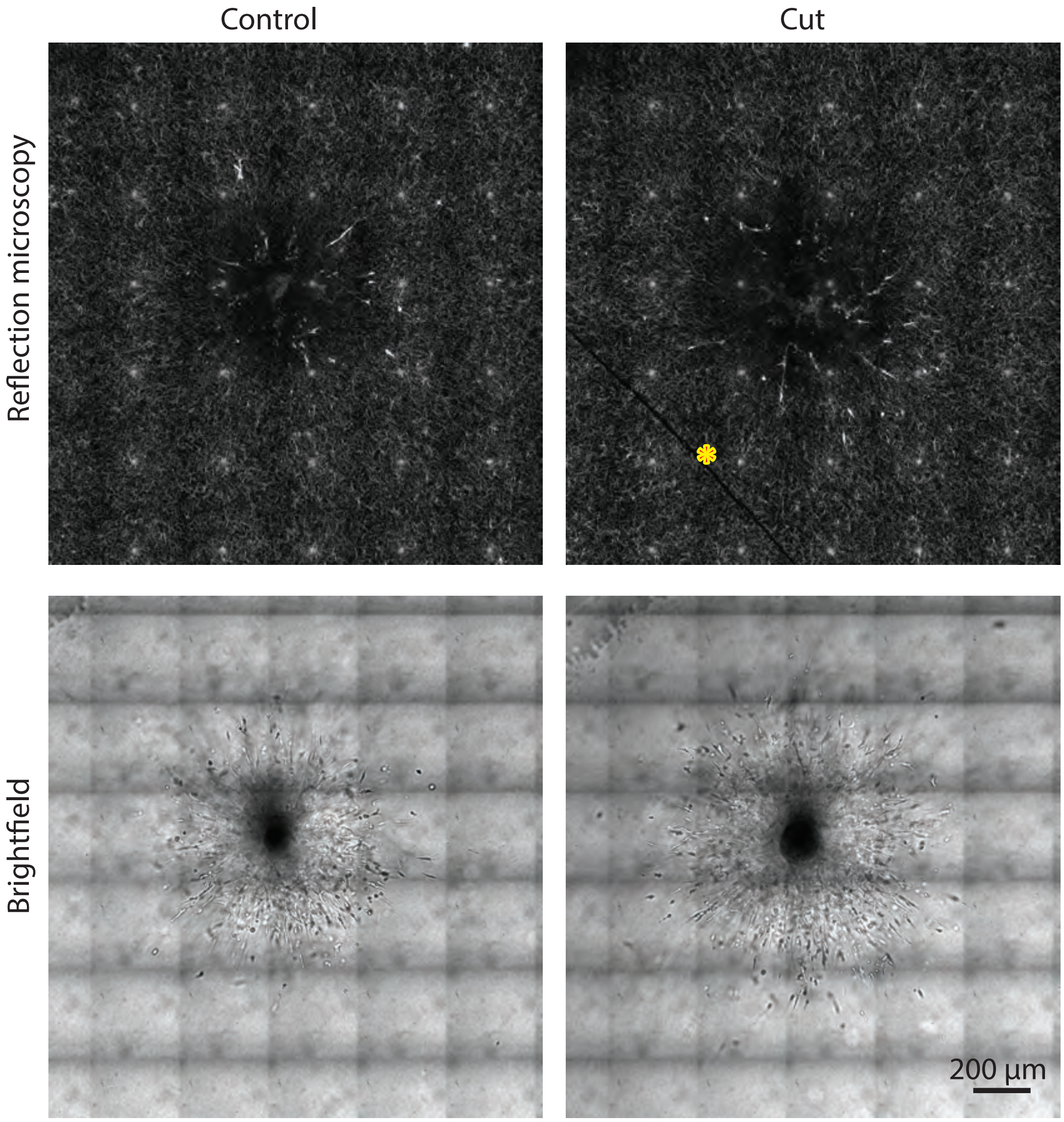

balcioglu supplementary figure S5
